# Pulmonary and Extra-Pulmonary Clinical Manifestations of COVID-19

**DOI:** 10.3389/fmed.2020.00526

**Published:** 2020-08-13

**Authors:** Kemmian D. Johnson, Christen Harris, John K. Cain, Cicily Hummer, Hemant Goyal, Abhilash Perisetti

**Affiliations:** ^1^Department of Internal Medicine, Louisiana State University Health Sciences Center, New Orleans, LA, United States; ^2^Department of Nurse Anesthesia, Texas Christian University, Fort Worth, TX, United States; ^3^College of Medicine, Rocky Vista University College of Osteopathic Medicine, Parker, CO, United States; ^4^Department of Internal Medicine, The Wright Center for Graduate Medical Education, Scranton, PA, United States; ^5^Department of Gastroenterology and Hepatology, University of Arkansas for Medical Sciences, Little Rock, AR, United States

**Keywords:** COVID-19, SARS-CoV-2, coronavirus 2019, COVID-19, pneumonia, SARS-CoV-1, MERS-CoV

## Abstract

The severe acute respiratory syndrome coronavirus−2 (SARS-CoV-2) has been recently identified as the culprit of the highly infectious, outbreak named coronavirus disease 2019 (COVID-19) in China. Now declared a public health emergency, this pandemic is present in more than 200 countries with over 14 million cases and 600,000 deaths as of July 18, 2020. Primarily transmitted through the respiratory tract, the most common clinical presentations of symptomatic individuals infected with SARS-CoV-2 include fever, dyspnea, cough, fatigue, and sore throat. In advanced cases, patients may rapidly develop respiratory failure with acute respiratory distress syndrome, and even progress to death. While it is known that COVID-19 manifests similarly to the 2003 Severe Acute Respiratory Syndrome (SARS) and the 2012 Middle East Respiratory Syndrome (MERS), primarily affecting the pulmonary system, the impact of the disease extends far beyond the respiratory system and affects other organs of the body. The literature regarding the extrapulmonary manifestations (cardiovascular, renal, hepatic, gastrointestinal, ocular, dermatologic, and neurological) of COVID-19 is scant. Herein, we provide a comprehensive review of the organ-specific clinical manifestations of COVID-19, to increase awareness about the various organs affected by SARS-CoV-2 and to provide a brief insight into the similarities and differences in the clinical manifestations of COVID-19 and the earlier SARS and MERS.

## Introduction

Over the last two decades, the coronavirus family has been identified as the source of several highly pathogenic global outbreaks. Some of the most notable are the 2003 Severe Acute Respiratory Syndrome coronavirus-1 (SARS-CoV-1), which caused the Severe Acute Respiratory Syndrome (SARS) outbreak in China, and the 2012 Middle East Respiratory Syndrome coronavirus (MERS-CoV) which caused the MERS outbreak in Saudi Arabia (1, 2). The most recent coronavirus outbreak likely developed in a local market (“wet market”) in China in December 2019 as a series of acute respiratory disorders [acute hypoxic respiratory failure, pneumonia, acute respiratory distress syndrome (ARDS) (3, 4)]. The causative pathogenic agent was found to be an enveloped, non-segmented, positive-sense RNA β-coronavirus, now termed Severe Acute Respiratory Syndrome coronavirus-2 (SARS-CoV-2). The disease is referred to as the coronavirus disease 2019 or COVID-19 (3).

The most commonly affected organ system by COVID-19 is the pulmonary system, with the most frequent clinical manifestations including cough, dyspnea, fever, and sore throat, similar to SARS and MERS (5, 6). In the severe disease state, the patient's clinical course is complicated by the development of pneumonia with ARDS, acute hypoxic respiratory failure, and/or death (7). While the pulmonary system is most commonly affected, extrapulmonary organs and organ systems (including the cardiac, gastrointestinal, hepatic, renal, ocular, and dermatologic) are also affected by COVID-19, which could have significant health consequences.

**Figure 1:**
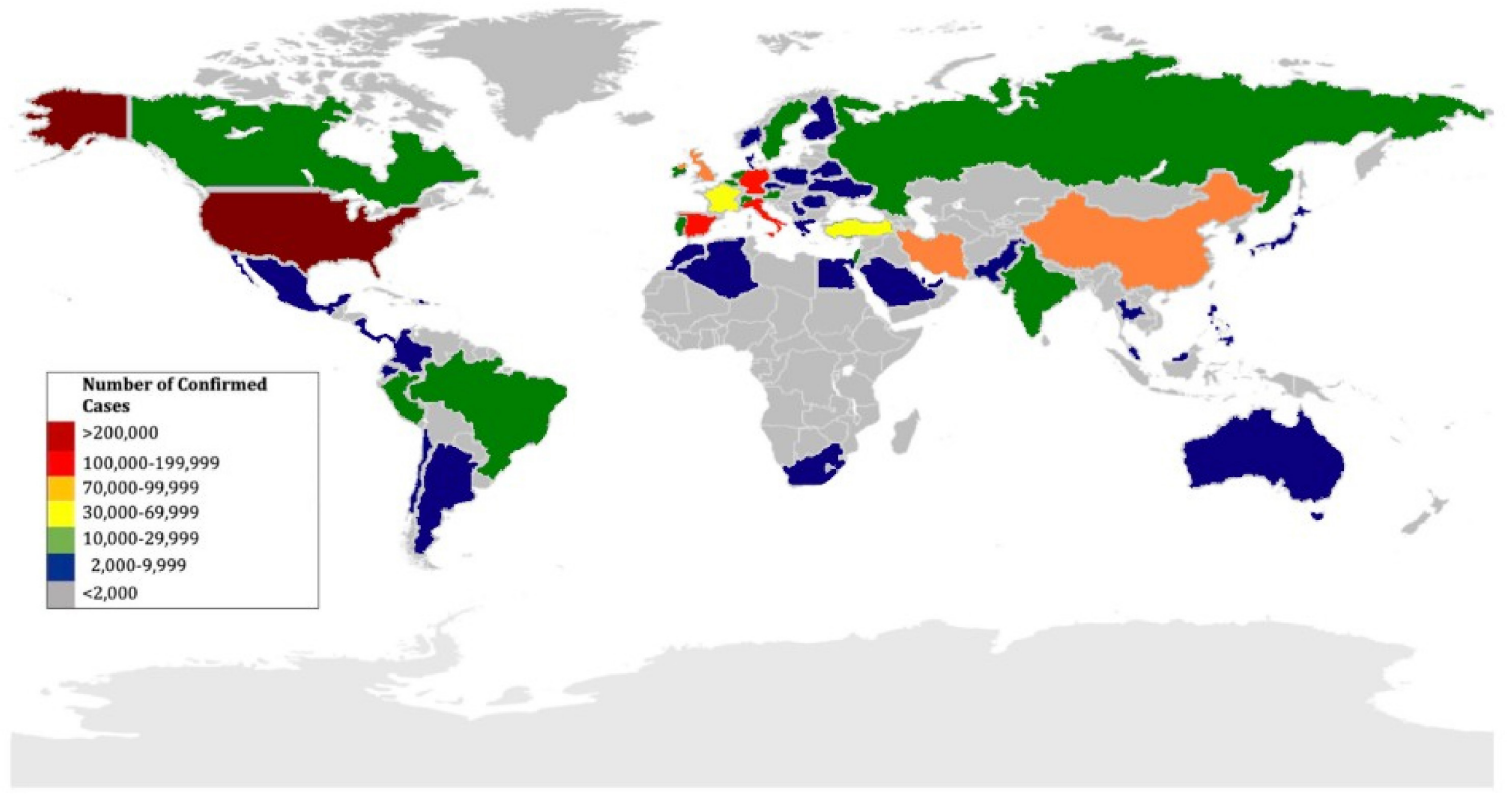
Geographic distribution of countries affected by COVID-19 as of April 16, 2020.

COVID-19-related mortality has affected more individuals than its antecedents, SARS and MERS, combined. The number of identified cases is steadily growing, and the outbreak has rapidly spread to many different areas in China and more than 200 other countries in a short period of time (Figure 1). As of July 18, 2020, 14 million cases and 600,000 deaths have been documented globally across over 200 countries and territories (8). Therefore, understanding the clinical manifestations of COVID-9 is crucial.

**Figure 2:**
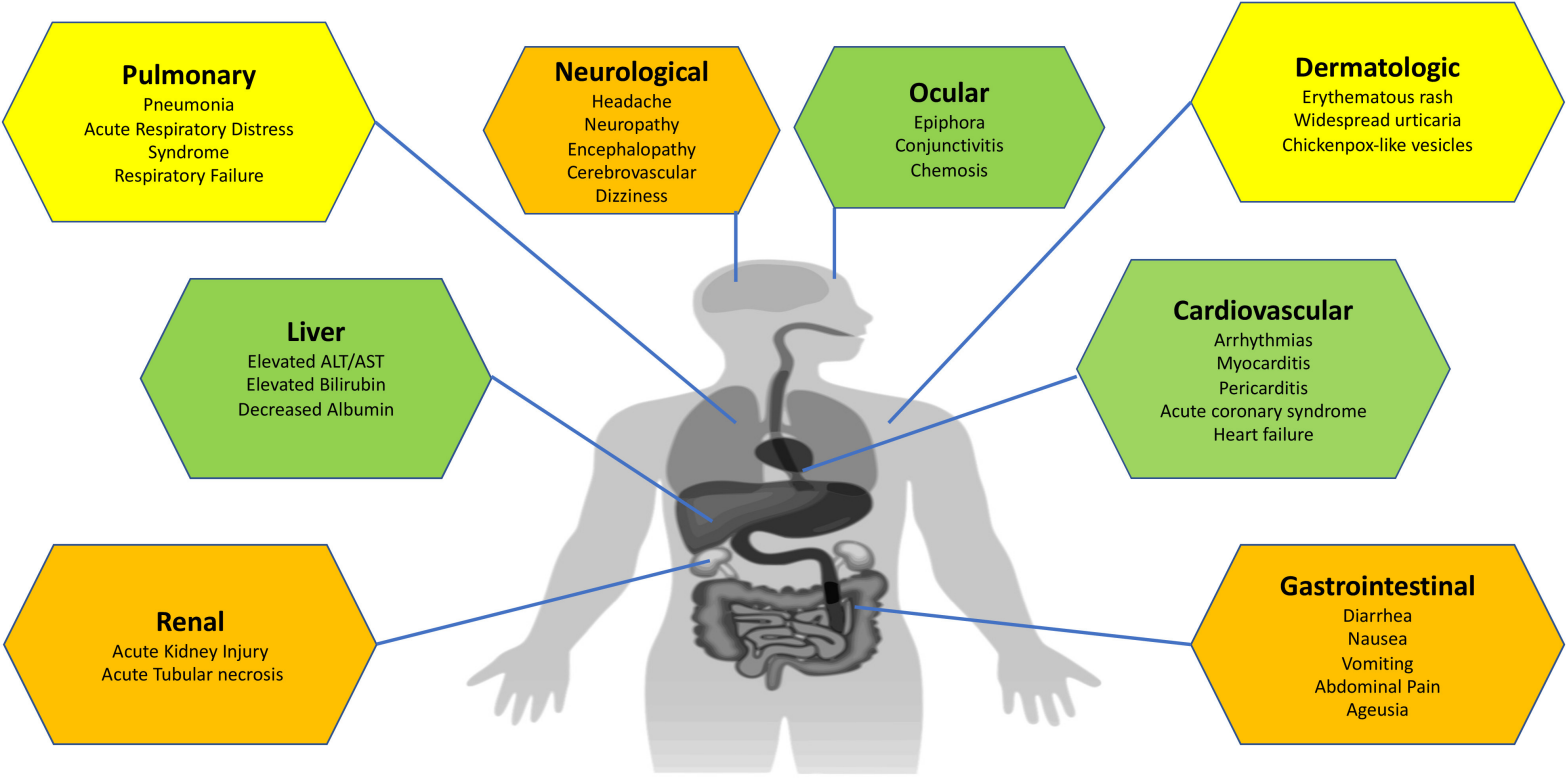
Organ-specific clinical manifestations of COVID-19.

In this article, we summarize the clinical manifestations of COVID-19 with an organ system-based approach to educate healthcare practitioners about both common and uncommon presentations of COVID-19 and to stay vigilant about this new disease (Figure 2).

## Pulmonary System

By far, the pulmonary system is the most common organ system affected by SARS-CoV-2. Several retrospective studies have consistently reported pulmonary manifestations in patients with COVID-19, which include cough, shortness of breath, sputum production, respiratory failure, and ARDS (Table 1) (5, 7, 9–17). In one large study (*n* = 1,099) from China, Guan et al. reported that 67.8% of patients with COVID-19 presented with cough, while 33% had sputum production, and 18.7% experienced shortness of breath (9). Similarly, another study (*n* = 262) of patients in Beijing demonstrated that cough occurred in almost half (45.8%) of patients with COVID-19, and dyspnea occurred in nearly 7% of patients (18). Multiple studies conducted in various countries have also demonstrated similar findings, showing that cough is the predominant pulmonary symptom in patients with COVID-19 (10, 19, 20). The main reason for the development of these symptoms is the presence of severe pneumonia in COVID-19 patients. However, the pulmonary symptoms can vary in COVID-19 patients, possibly due to variation in severity of disease at the time of presentation. In a study (*n* = 41) by Huang et al. on patients with confirmed SARS-CoV-2 infection, the most common symptoms were fever (98%) followed by cough (76%), with over half (55%) of the patients developing dyspnea (5).

**Table 1:** Published meta-analyses of Pulmonary Manifestations of COVID-19.

**Authors**	**Number of studies**	**Number of cases**	**Fever**	**Cough**	**Dyspnea**	**Sputum production**	**Sore throat**
Long-quan Li et al.	10	1995	88.50%	68.60%	21.90%	28.20%	NR
Rodriguez-Morales et al.	19	656	88.70%	57.60%	45.60%	NR	11.00%
Yang et al.	8	46248	91.0%	67%	30.00%	NR	NR
Cao et al.	31	46,959	87.30%	58.10%	38.30%	NR	12.00%
Borges et al.	61	59,254	82.00%	61.00%	26%	NR	10.00%
Sun et al.	10	50,466	89.00%	72.20%	NR	NR	NR
Fu et al.	43	1,600	83.30%	60.30%	24.90%	26.90%	12.30%
Di Mascio et al.	19	79	82.60%	57.10%	27.00%	NR	NR

ARDS is a known severe pulmonary complication of COVID-19, where patients experience severe hypoxia refractory to oxygen therapy (9). Further, COVID-19 patients with severe pneumonia can deteriorate and develop life-threatening acute respiratory failure and ARDS, requiring intensive medical care. A metanalysis (*n* = 656) of observational studies and case reports showed that nearly one third (32.8%) of patients with COVID-19 developed ARDS during their hospital admission (7). Similarly, in a retrospective analysis of clinical findings in 85 patients with confirmed COVID-19, 74.1% of patients developed ARDS during their hospitalization (21). Lai et al. (*n* = 72) identified that about 20% developed ARDS and >25% of patients with COVID-19 required intensive care unit (ICU) admission (22). In one large retrospective study (*n* = 710) by Yang et al., 61.5% of patients with COVID-19 pneumonia died in 28 days with a mean interval from ICU admission to death being 7 days (23).

### Comparison of Pulmonary Manifestations Among Covid-19, SARS, and MERS

Given that SARS-CoV-1, SARS CoV-2, and MERS-CoV are all members of the coronavirus family, several comparisons have been made regarding the pulmonary manifestations of these diseases (Table 2). The respiratory manifestations of SARS and MERS are very similar to COVID-19. The most common presenting symptoms in patients with COVID-19, SARS, and MERS are cough and dyspnea. Further, in patients with COVID-19, dry cough is present in the early stage of infection, progressing to an expectorant cough with the growing severity of the illness (24). Similarly, in patients with SARS, initial symptoms included cough (61.8%) and dyspnea (40.8%) (24). There are fewer upper airway symptoms that occur in COVID-19 as compared to SARS (25), while patients with MERS may present with hemoptysis, cough, and shortness of breath.

**Table 2:** Comparison of coronavirus outbreaks between SARS, MERS, and COVID-19.

	**SARS**	**MERS**	**COVID-19**
Year outbreak	2003	2012	2019
Source of outbreak	Bats	Camels, camel products	Bats, seafood
Outbreak location	Guangdong, China	Saudi Arabia	Wuhan, China
Route of transmission	Droplets, contact	Contact	Droplets, contact
Case fatality rate	9.5%	35%	Not yet determined
Basic reproduction number, R0	4.0	1.0	2.0–3.5

### Putative Mechanisms of Pulmonary Injury

**Figure 3:**
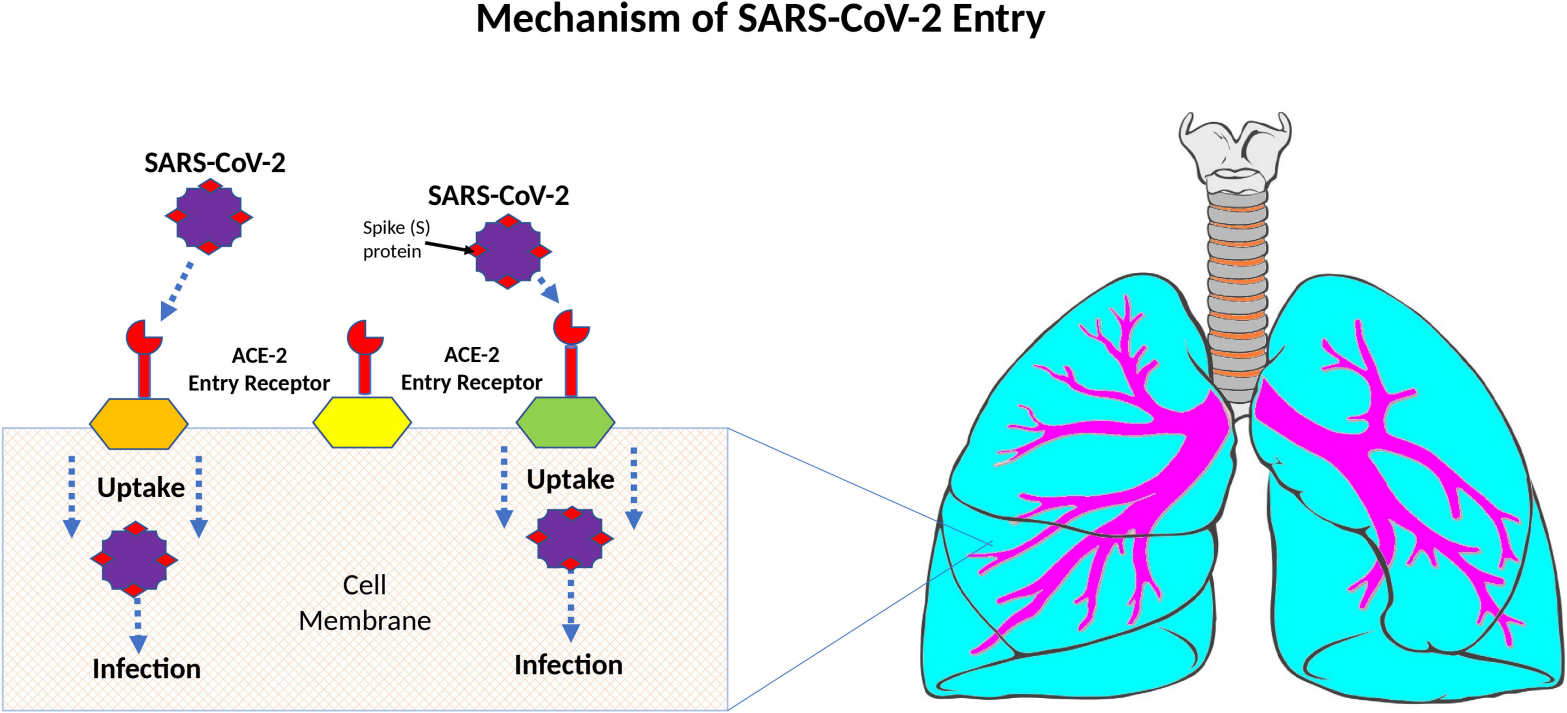
Mechanisms of SARS-CoV-2 entry.

It has been well-established that the target of entry for SARS-CoV-2 is the angiotensin-converting enzyme-2 (ACE-2) receptors (Figure 3) (3, 26, 27). ACE-2 receptors are expressed in type I & II alveolar cells, and airway epithelial cells (25). The virus enters these cells using cell-mediated endocytosis and starts a cascade of pro-inflammatory cytokines such as interleukin (IL)-1β, IL-6, and tumor necrosis factor (TNF) (28). When SARS-CoV-2 binds the ACE-2 receptor, it reduces the expression of ACE-2 (29). Interestingly, SARS-CoV-1 and SARS-CoV-2 bind the same ACE-2 receptor; however, SARS-CoV-2 binds this receptor with 10–20 times greater affinity than SARS-CoV-1 (26).

## Gastrointestinal Manifestations of COVID-19

Though respiratory symptoms predominate, gastrointestinal (GI) complications from SARS-CoV-2 infection have also been described, and may even precede respiratory symptoms (10, 30). The most frequently reported GI manifestation include nausea, vomiting, diarrhea, and abdominal pain (10, 31–36). In a retrospective study (*n* = 138) of hospitalized COVID-19 patients, 10% of patients reported both nausea and diarrhea. Abdominal pain and vomiting were recorded in 5 and 3% of patients, respectively (10). Of note, 10% of patients experienced nausea and diarrhea between 1 and 2 days before experiencing respiratory symptoms, suggesting that GI symptoms may atypically present as one of the initial clinical manifestations of COVID-19 (10). Similarly, in a large metanalysis of 10 studies with a total of 1995 cases, diarrhea occurred in 4.8% of cases, while nausea and vomiting occurred in 3.9% of cases (10). Jin et al. also recorded either diarrhea, nausea, or vomiting in 74 of the 651 infected patients reviewed in their study (32). Interestingly, patients who experienced GI symptoms were more likely than those without GI symptoms to have a more severe disease course, characterized by greater degrees of liver insult (17.57 vs. 8.84%), development of ARDS (6.76 vs. 2.08%), and ICU admission requiring mechanical ventilation (6.76 vs. 2.08%) (32). Further, nearly a quarter (22.97%) of the study population who experienced critical illness reported GI symptoms at initial presentation (32).

### Possible Mechanisms of GI Manifestations

The fecal-oral route has been proposed as a potential mechanism of GI infection with SARS-CoV-2 (37–39) due to the identification of SARS-CoV-2 RNA in the stool specimens of infected patients (40). Xiao et al. studied the RNA in feces from 73 patients with COVID-19, and 53% of the patients tested positive for SARS-CoV-2 RNA in the stool (41). Additionally, studies have found overexpression of ACE-2 in the epithelial cells of the GI tract, suggesting SARS-CoV-2 replication in the GI tract (42). A case of positive fecal specimen in a symptomatic COVID-19 patient with a negative pharyngeal and sputum specimen has also been published in the literature (43).

## Hepatic Manifestations of COVID-19

The liver is another organ which can be affected by SARS-CoV-2 (6, 44–46). Commonly reported hepatic manifestations of COVID-19 include elevations in serum levels of alanine transaminase (ALT), aspartate transaminase (AST), and bilirubin, while levels of albumin are decreased (6, 44). In a single-center retrospective study (*n* = 99) in patients with reverse transcription-polymerase chain reaction (RT-PCR) confirmed COVID-19, nearly half (43%) of patients demonstrated abnormal liver chemistries (6). Decreased albumin was noted in 98% of patients, while serum levels of AST, ALT, and bilirubin were elevated in 35, 28, and 18% of patients, respectively. Similarly, in an analysis of 1,099 patients, increased levels of AST were observed in 18.2% of patients with non-severe disease and 39.4% of patients with severe disease, while increased ALT levels were observed in 19.8% of patients with non-severe disease and 28.1% of patients with severe disease (9). The authors in this study used the 2007 American Thoracic Society criteria for community-acquired pneumonia to define COVID-19 disease severity (47). With these results, it appears that the degree of liver injury may be associated with COVID-19 disease severity. In a recent meta-analysis Lippi et al., demonstrated that hepatic factors that were predictive of patients with an unfavorable course of COVID-19 requiring ICU admission included an increase in levels of ALT (1.5–1.8-fold), AST (1.8-fold), total bilirubin (1.2–1.3-fold) and decreased albumin (0.8-fold) (48).

Other studies have demonstrated isolated elevations in AST alone. In a study (*n* = 81) by Shi et al., more than 50% of COVID-19 patients were observed to have elevated levels of AST with normal ALT (44). Similarly, in another study (*n* = 41), 63% of ICU admitted COVID-19 patients had elevated AST vs. only 25% of patients who did not require ICU care (5).

Patients infected with the 2003 SARS-CoV-1 also experienced liver impairment (49–52). Similar to the hepatic injuries associated with COVID-19, the most frequent GI clinical manifestations of SARS included elevations in levels of serum bilirubin, ALT and/or AST, and decreased levels of serum albumin (49–52).

### Mechanisms of Hepatic Infection/Injury

The number of studies to better understand the mechanisms of hepatic injury in patients infected with coronaviruses is limited. One proposed mechanism for hepatic injury with SARS-CoV-1 includes heightened inflammatory response to the virus infection (53). This mechanism has been supported by the abnormally high serum levels of cytokines (serum IL-1, IL-6, IL-10) observed in SARS patients and deranged liver chemistries (53). Another proposed mechanism is direct hepatic injury by SARS-CoV-1 via the entry of the ACE-2 receptor on hepatic endothelial cells. Further, SARS-CoV-1 viral particles have also been identified in the liver autopsies of deceased SARS patients and SARS viral genomes have been found by RT-PCR in hepatocytes (54). Contrarily, the proposed mechanism of hepatic injury with MERS-CoV involves dipeptidyl peptidase-4 (DPP-4) as its entry receptor to establish infection in the hepatocytes (55). Several animal and human studies have demonstrated higher DPP-4 expression in liver, and MERS-CoV can infect the liver cells via DPP-4 on the cell surface, causing cell damage and mild to moderate liver injury (56).

Data regarding the mechanism of hepatic injury by SARS-CoV-2 is scarce. It has been proposed that SARS-CoV-2 attaches to ACE-2 as its entry receptor, similar to SARS-CoV-1. A preliminary study by Chai et al. showed over expression of ACE-2 specifically in cholangiocytes, indicating that the virus may potentially bind to cholangiocytes to cause hepatic dysfunction (57). Further, liver biopsy of a deceased COVID-19 patient with deranged liver chemistries showed moderate microvascular steatosis, and mild lobular and portal activity, which was thought to be caused by SARS-CoV-2 infection. However, more studies are needed to further evaluate the mechanism of injury. It is important to mention that concurrent use of hepatotoxic medications in patients with COVID-19 may contribute to liver injury in patients with COVID-19 who are receiving treatment, complicating the discovery of the exact etiology of liver injury (58).

## Cardiovascular Manifestation of COVID-19

**Figure 4:**
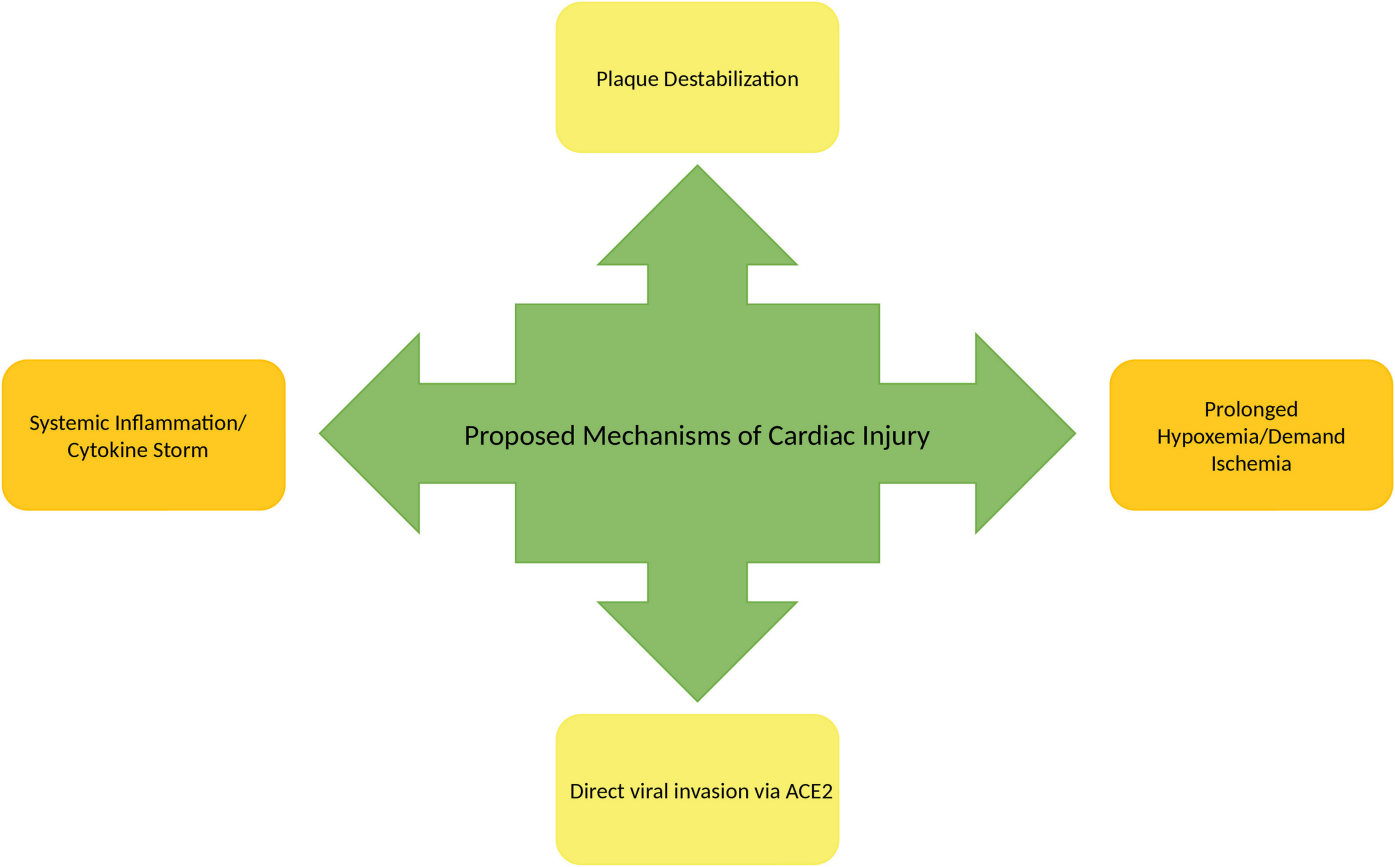
Mechanisms of cardiac injury in COVID-19.

The cardiac manifestations of COVID-19 include cardiac arrhythmias, myocarditis, pericarditis, acute coronary syndrome (ACS), heart failure, cardiogenic shock, and cardiac arrest (Figure 4). Though there appears to be no difference in the prevalence of cardiovascular disease (CVD) amongst those with COVID-19 compared to the general population, patients with pre-existing CVD are at higher risk of developing severe COVID-19 (59, 60). A meta-analysis of COVID-19 patients revealed that the prevalence of hypertension, cardio-cerebrovascular disease, and diabetes mellitus was 17.1, 16.4, and 9.7%, respectively. Moreover, the prevalence of hypertension, cardio-cerebrovascular disease, and diabetes mellitus was 2-, 3-, and 2-folds in severe/ICU cases as compared to non-ICU cases, respectively. Additionally, the meta-analysis acknowledged and analyzed viral damage to the heart noting that at least 8% of patients with COVID-19 suffered acute cardiac injury (59). The meta-analysis also evaluated the incidence of myocardial injury in severe/ICU cases and non-ICU cases. Acute cardiac injury was assessed using cardiac markers troponin I/T or CK if troponin I/T were not provided (59). The analysis showed a 13-fold higher incidence of myocardial injury as measured by elevations in Troponin I/T or CK in severe/ICU cases compared to non-ICU COVID-19 cases (59). Additionally, in a separate study (*n* = 187), Guo et al. showed that mortality in COVID-19 patients during hospitalization was greatly associated with presence of CVD and myocardial injury. The study revealed inpatient mortality of 7.62% for patients without underlying CVD and normal troponin T (TnT) levels, 13.33% for those with underlying CVD and normal TnT levels, 37.50% for those without underlying CVD but elevated TnT levels, and 69.44% for those with underlying CVD and elevated TnTs (61). This data indicate that those with cardiovascular comorbidities are more likely to have a poor outcome with a more severe COVID-19 course. Similarly, previous SARS outbreaks had increased mortality associated with CVD and diabetes in SARS (59).

In addition to the myocardial injury evidenced by elevations in troponins, another cardiac manifestation of COVID-19 includes arrythmias. In a retrospective study (*n* = 137) by Lui et al., heart palpitations were reported as an initial symptom in 7.3% of patients with COVID-19 (19). Similarly, 17% of hospitalized COVID-19 patients had unspecified arrhythmias in a separate case series(*n* = 138) (10). Another study (*n* = 187) reported ventricular tachycardia/ventricular fibrillation at a rate of 5.9% in hospitalized COVID-19 patients in Wuhan, China (61). Though there has been no biopsy or cardiac magnetic resonance imaging (cMRI) proven fulminant myocarditis or pericarditis, several case series and case reports recognize these as one of the manifestations of COVID-19 based on clinical suspicion and objective data (60, 62). Additionally, development of heart failure or cardiogenic shock was observed in several studies. In a retrospective cohort study of 191 hospitalized COVID-19 patients at two Chinese hospitals, 23% of patients had evidence of heart failure or cardiogenic shock (63).

### Mechanisms of Development of Cardiac Manifestations

Though the exact mechanism of myocardial injury, development of heart failure, and cardiogenic shock is unknown, there are a number of proposed mechanisms to consider. One of those mechanisms involves direct cardiac myocyte toxicity associated with viral invasion. Similar to SARS-CoV-1, SARS-CoV-2 has binding affinity for the ACE-2 receptor in myocardial cells. Zou et al. performed mapping of cells in various organ systems expressing ACE-2 with the use of single-cell RNA sequencing. Cells expressing similar or more ACE-2 than lung type II alveolar cells (AT2) were deemed as having the potential for increased vulnerability to SARS-CoV-2 (64). In their study, >7.5% of myocardial cells displayed ACE-2 expression suggesting that the heart may be at high risk for direct cellular toxicity by SARS-CoV-2 entry and replication. This ability to infiltrate cardiac tissues appears to be similar to MERS-CoV and SARS-CoV-1. In an animal model study using transgenic mice, MERS-CoV RNA was detectable in the heart (65). Similarly, in a study from the Toronto SARS outbreak, RNA of SARS-CoV-1 was found in 35% of cardiac tissues on autopsy (66).

Another proposed mechanism of cardiac effects of COVID-19 is heightened release of pro-inflammatory cytokines through activation of the innate and adaptive immune system. The increased production of cytokines such as IL-6, IL-10, and TNF-α can lead to multiorgan failure. In the past, IL-6 has been associated with cardiomyopathy. Additionally, this inflammatory state can promote and contribute to atherosclerotic plaque rupture (63) and acute coronary syndrome. In the setting of critical illness due to COVID-19, prolonged exposure to catecholamines and cytokine storm as a response to infection can result in myocardial damage as well as stress-induced cardiomyopathy (59).

Due to the respiratory sequelae of COVID-19, some patients suffer from hypoxemia which is another proposed mechanism of cardiac injury. As prolonged hypoxemia results in reduced cellular capacity to metabolize aerobically, cells are subsequently switched to anaerobic metabolism. Anaerobic metabolism produces a more acidotic state intracellularly due to increased lactic acid production. Subsequently, increased free radical production and direct destruction of phospholipid cell membranes occur (59). Hypoxemia can also increase calcium ion influx which may lead to cardiac myocyte apoptosis (59). Demand ischemia associated with critical illness can produce similar mechanisms of cardiac injury.

Though more research is needed to further assess the pathogenesis associated with COVID-19 myocardial injury, data obtained thus far indicates the presence of viral-related heart damage. This damage manifests in a variety of ways including evidence of arrythmias, pericarditis, myocarditis, heart failure, cardiac shock, and cardiac arrest (59, 61). Finally, current data suggests cardiovascular disease, cardiac manifestations, and cardiac injury in the setting of COVID-19 are clinically relevant predictors of overall disease severity and mortality (59, 61).

## Renal Manifestations of COVID-19

Another organ system affected by SARS-CoV-2 is the renal system with development of acute kidney injury (AKI). This can occur in those with chronic kidney disease (CKD) as well as those with no evidence of prior renal impairment. Though acknowledged as a rare occurrence in SARS, it appears that AKI may be more common in COVID-19 (67, 68). In a single center case series (*n* = 138) assessing clinical characteristics of patients with COVID-19, Wang et al. noted that ~4% of these patients with COVID-19 had an AKI (10). Huang et al. determined in a separate study of 41 COVID-19 positive patients that ~7% had evidence of an AKI (69). In a small Washington state study consisting of 21 critically ill COVID-19 patients, 19.1% (*n* = 6) had acute kidney failure according to KDIGO guidelines (70). Analysis of 51 critically ill COVID-19 patients in a Wuhan, China study showed 29% (*n* = 15) developed an AKI (23). Similarly, during the SARS outbreak, AKI was also observed. In a study performed by Chu et al., 6.7% of 537 patients with SARS developed an AKI in the setting of normal Cr on admission (67). Additionally, Chu et al. revealed a significantly higher mortality rate (91.7%) associated with those having evidence of renal impairment compared to those with normal renal function in the setting of SARS (8.8%). In Wang's study of COVID-19 patients, AKI was observed more in ICU than non-ICU patients. This might indicate that severity of illness progression associated with COVID-19 may be significantly impacted by the presence of renal impairment.

### Mechanism of Injury

Several potential pathophysiological explanations have been suggested to explain renal impairment in COVID-19. ACE-2 receptor have been shown to be highly expressed in the proximal tubules and urothelial cells of the bladder on single cell RNA sequencing (63). The increased susceptibility of the kidney to viral entry associated with ACE-2 expression make it a possible target for direct cellular toxicity. Moreover, SARS-CoV-2 has a significantly higher affinity for the ACE-2 receptor which could explain the higher incidence of AKI in COVID-19. Another possible mechanism of AKI in COVID-19 is significantly higher immune response to infection and multiorgan failure. SARS-CoV-2 induces the release of inflammatory cytokines IL-2, IL-7, IL-10 which are believed to be involved in the pathology of AKI (71). Additionally, in critically ill patients, the presence of hypovolemia, rhabdomyolysis, hypoxemia, sepsis, and septic shock associated with this viral illness are likely to contribute significantly to renal impairment. Furthermore, the possibility that the etiology of AKIs seen in COVID-19 patients is multifactorial should also be considered.

## Neurological

**Figure 5:**
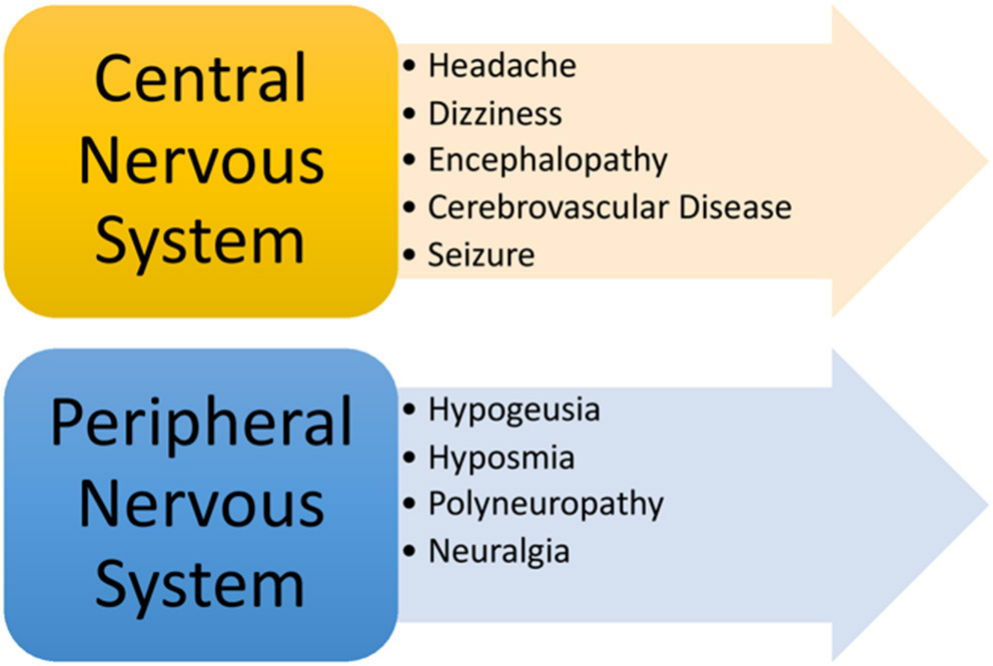
Neurological manifestations of COVID-19.

More recently, a wide range of neurological complications have been reported in patients with COVID-19 suggesting that SARS-CoV-2 may affect both the central and peripheral nervous system (Figure 5). Commonly reported central nervous system (CNS) manifestations include headache, acute cerebrovascular disease, dizziness, and encephalopathy. In a retrospective study (*n* = 214) of confirmed COVID-19 patients, 36.4% of subjects collectively experienced either dizziness, headache, cerebrovascular disease, and/or reduced consciousness (72). Headache appears to be one of the most common CNS symptoms, which has been reported at a rate of 6–13% in patients with COVID-19 (5, 6, 23, 73). Dizziness occurred in nearly 9–17% of patients based on recent studies (10, 72), while reduced levels of consciousness and confusion occurred in 7.5 and 9% of COVID-19 patients, respectively (6). In a recent case series (*n* = 58) of COVID-19 patients who developed ARDS, several neurologic findings, including encephalopathy, confusion, agitation and corticospinal tract signs were reported in 84% of cases. However, it is unclear whether or not these neurological signs and symptoms were directly related to infection with SARS-CoV-2, medication withdrawal, or cytokine effects (74). To a lesser extent, seizure has also been reported in a minority of cases (0.5%) (72). Similarly, rare cases of confirmed viral encephalitis and meningitis have been described in small case reports of patients with SARS-CoV-2 detected in the cerebrospinal fluid (75, 76).

Cerebrovascular disease represents another cluster of CNS manifestations of COVID-19 that have been cited in the literature. In a retrospective, observational study (*n* = 221) of patients with COVID-19 in Wuhan China, acute ischemic stroke (confirmed on head CT) was reported in 5% of subjects. Additionally, cerebral venous sinus thrombosis (confirmed with CT venography), and cerebral hemorrhage both occurred at a rate of 0.5% in this cohort (77). Another study demonstrated that ischemic stroke and cerebral hemorrhage (both confirmed on head CT) were collectively noted in 2.8% of 214 patients with COVID-19 (72). Even further, one case report described a case of acute necrotizing hemorrhagic encephalopathy associated with COVID-19 in a middle aged female who presented with acute encephalopathy (78).

Based on recent studies, neurologic manifestations appear to occur more frequently in patients with severe disease courses (79). Mao et al. demonstrated that neurological manifestations (specifically cerebrovascular disease, reduced consciousness and myopathy) occurred more frequently (45.5 vs. 30%) in patients with severe disease compared to those with non-severe disease. Of note, those with more severe disease (as defined by the previously described American Thoracic Society guidelines) were also noted to have a greater burden of co-morbidities. However, it is unclear whether these neurologic manifestations hold any prognostic value in regards to COVID-19 mortality (72).

In addition to CNS manifestations, peripheral nervous system (PNS) findings related to COVID-19 have been described in the literature. Clinical data has demonstrated that patients with COVID-19 may experience changes in smell and/or taste, in addition to polyneuropathy and/or even neuralgia. In a retrospective study of COVID-19 patients, hypogeusia, and hyposmia occurred in 5.6 and 5.1% of patients, respectively, while neuralgia or peripheral nerve pain occurred in 2.3% of the study patients. In the same study, changes in smell occurred in 12% of patients prior to any respiratory symptoms (80), suggesting that hyposmia may uncommonly precede development of any respiratory symptoms. Other studies have demonstrated similar findings (72, 81). Another study (*n* = 60) demonstrated that reduced smell function was prominent (98%) among patients diagnosed with COVID-19, but concluded that changes in smell did not hold any prognostic value (82). In addition to changes in smell, one study showed that up to 88% of patients with COVID-19 experienced changes (diminished or complete loss) in taste before and during their disease course (83). As such, it is reasonable to consider change in smell and/or taste as potential warning signs for COVID-19, while neuralgia and peripheral neuropathy may be considered disease manifestations that may along the course of infection. Clinicians should conduct a thorough neurological history and physical to identify early signs and symptoms of patients who many warrant COVID-19 testing (83).

## Ocular/Ophthalmic

Healthcare professionals can get exposed to ocular secretion of the infected patients and these secretions could become a fomite for viral spread. Ocular manifestations such as conjunctivitis, retinitis, anterior uveitis, and optic neuritis have been reported due to infections from the coronaviruses in the past (84–86). However, there is paucity of literature regarding the ocular manifestations of COVID-19, possibly because these manifestations are under-recognized and under-reported.

In a case series of 36 patients with confirmed COVID-19, nearly one third (31.6%) of patients developed ocular manifestations such as chemosis, epiphora, and conjunctival congestion. Interestingly patients with ocular manifestations experienced a severe disease course. Loon et al. published a case series of patients with suspected and probable SARS infection who had tear samples collected and analyzed by PCR. Using WHO case definitions of suspected and probable cases, eight patients were classified as probable SARS (based on chest imaging suggestive of pneumonia or ARDS) and 28 were classified as suspects of SARS (anyone experiencing fever >100.4°F, respiratory symptoms and known contact with a confirmed case of SARS) (87). Of 36 subjects tested, three with probable SARS had positive SARS-CoV results from their tear samples suggesting that SARS-CoV-1 can exist in tears and may potentially be a source of spread among healthcare workers and inoculating patients (88). Similarly, another earlier predecessor of the SARS-CoV-2, the human CoV-NL63 virus was isolated from nasopharyngeal aspirate from an infant who had conjunctivitis and bronchiolitis (89). Another retrospective study of 18 children with acute respiratory tract infection due to CoV-NL63 showed that three patients also developed conjunctivitis (90). However, some controversy endures as some authors have proclaimed that ACE-2 receptors predominantly exist in the posterior eye, which would not account for the cases of anterior uveitis and conjunctivitis related to SARS-CoV-1 (91).

## Cutaneous Manifestations of COVID-19

Cutaneous findings have also been reported as a manifestation of COVID-19. While there is little data regarding the topic, currently reported manifestations include erythematous rash, vesicular lesions, and urticaria. In a small analysis (*n* = 88) of patients who tested positive for COVID-19, nearly 20% of patients developed skin findings (92). Of the 88 positive patients, eight presented with skin findings at disease onset, while 10 developed skin findings during hospitalization. Nearly 16% developed an erythematous rash, while 1.1 and 3.4% developed vesicular lesions and urticaria, respectively. The most commonly affected cutaneous region was the trunk and most lesions resolved within a few days (92). While preliminary data exists, many more studies are needed to provide additional information regarding the dermatologic manifestations of COVID-19.

### Asymptomatic Covid-19 Patients

While SARS-CoV-2 has been shown to affect various organs with a variety of clinical manifestations, some patients with RT-PCR detected SARS-CoV-2 remain completely asymptomatic. A number of studies report a wide incidence rate of asymptomatic infections, ranging from 1.6 to 56.6% (93–98). According to these studies, asymptomatic patients typically experience none of the aforementioned clinical signs and/or symptomology. Even further, this subgroup of patients have little to no abnormalities on radiological imaging. While some with asymptomatic infection may develop into symptomatic cases, most progress without clinical deterioration. Hu et al. conducted a study (*n* = 24) in asymptomatic patients (no symptoms at the time of screening) who tested positive for COVID-19. Of the 24 patients in the study, mortality was not observed in any of the patients, however 20% later developed fever, cough, and/or fatigue during the course of hospitalization (99).

### Similarities and Differences in Comorbid Health Conditions in Covid-19, MERS, and SARS

It has been well-established that patients with pre-existing comorbidities generally experience worse health outcomes (higher rates of mortality, ICU admission, mechanical ventilation) compared to patients who do not have any underlying health conditions. This predisposition to more severe disease can be attributed to the negative impact that comorbidities have on the individual's immune system and subsequent decreased ability to fight infection (100). Prior to the emergence of COVID-19, previous studies have substantiated the notion that patients with SARS and patients with MERS who also had comorbidities generally experienced poorer health outcomes (101). These comorbidities commonly included heart disease, diabetes mellitus (DM), chronic obstructive pulmonary disease (COPD), cancer, chronic renal disease, hypertension, ischemic heart disease, congestive heart failure, asthma, and cerebrovascular accident (CVA). In an analytical study (*n* = 115) of patients with SARS, diabetes, and heart disease were each found to be independent risk factors for mortality. Specifically, patients with heart disease and/or diabetes conferred a 12.5-time higher risk of mortality (102). Another retrospective case series (*n* = 144) showed that SARS patients with a diagnosis of diabetes had a 3-fold increased the risk of death, ICU admission, or mechanical ventilation, while SARS patients with other comorbid conditions like COPD, heart disease or cancer had a 2.5 increased risk. In the same study, only one of the 144 subjects, a patient with no known comorbidities (former smoker), experienced mortality (103).

Similarly, in patients with MERS, pre-existing health conditions were shown to impact the severity of the disease course. In a 2013 Saudi Arabia study (*n* = 47) of patients with MERS, nearly 64% of the study population with diagnosed comorbidities (diabetes, hypertension, cardiac disease, and chronic renal disease) experienced mortality while only 14% of the study population without comorbidities died (104). Another study (*n* = 1,743), which investigated the impact of comorbidity on mortality rate in MERS patients, found that patients without any comorbidities had a higher 21-day survival rate compared to patients with known comorbidities. Further, MERS patients with comorbidities had a 4-fold risk for fatal health outcomes compared to those without comorbidities (105).

Similar to patients with MERS and SARS, disparities are seen between health outcomes of COVID-19 patients with pre-existing health conditions and those without (12). In a retrospective analysis (*n* = 138) of patients with COVID-19, nearly half (46.4%) of patients had an underlying health condition. Even further, those patients burdened with multiple comorbidities (72.2%) were more likely to require ICU admission compared to those with no comorbidities (37.3%) (10). While it is well-known that having comorbidities establishes an increased risk of disease severity, few studies have previously identified which conditions confer the greater risk of COVID-19 disease severity. In a large metanalysis (*n* = 1,558) of patients with COVID-19, hypertension, DM, COPD, heart disease, and cerebrovascular disease were all found to be independent risk factors for severe disease (defined by either an ICU admission or severity of symptoms), while patients with liver disorders, cancer, or kidney disease experienced no increased risk (106). In another metanalysis (*n* = 1,813), Jain et al. showed that patients with COVID-19 who also had underlying COPD, hypertension, and/or cardiovascular disease had a greater risk of requiring ICU admission or experiencing severe disease (107). Even further, COPD was shown to be the greatest predictive comorbid risk factor for severe disease and ICU admission followed by cardiovascular disease and hypertension (107).

## Conclusion

COVID-19 has become a pandemic and a public health emergency, affecting more individuals than previous coronavirus outbreaks with SARS and MERS. The clinical manifestations of COVID-19 are primarily related to the pulmonary system, and include dyspnea, cough with sputum production, fatigue and in severe cases, ARDS, respiratory failure, and even death. Extrapulmonary clinical manifestations of COVID-19 exist and affect multiple other organs and organ systems including cardiovascular, renal, hepatic, gastrointestinal, ocular, dermatologic, and neurological systems. Clinicians should be aware of the variable organ and organ systems affected in patients with COVID-19 and the potential disease course in patients.

## Author Contributions

KJ, CHa, JC, CHu, HG, and AP equally contributed to this paper with conception and design of the study, literature review and analysis, drafting and critical revision and editing, and final approval of the final version. All authors contributed to the article and approved the submitted version.

## Conflict of Interest

The authors declare that the research was conducted in the absence of any commercial or financial relationships that could be construed as a potential conflict of interest.
